# The role of hedgehog signaling in gastric cancer: molecular mechanisms, clinical potential, and perspective

**DOI:** 10.1186/s12964-019-0479-3

**Published:** 2019-11-27

**Authors:** Yan Xu, Shumei Song, Zhenning Wang, Jaffer A. Ajani

**Affiliations:** 1 Department of Gastrointestinal Medical Oncology, Unit 426, The University of Texas MD Anderson Cancer Center, 1515 Holcombe Boulevard, Houston, TX 77030-4009 USA; 2 Department of Surgical Oncology and General Surgery, First Hospital of China Medical University, 155 North Nanjing Street, Shenyang, 110001 People’s Republic of China

**Keywords:** Hedgehog signaling, Small-molecule inhibitor, Cancer stem cells, Gastric cancer, Targeted therapy

## Abstract

Patients with advanced gastric cancer usually have a poor prognosis and limited therapeutic options. Overcoming this challenge requires novel targets and effective drugs. The Hedgehog (Hh) signaling pathway plays a crucial role in the development of the gastrointestinal tract and maintenance of the physiologic function of the stomach. Aberrantly activated Hh signaling is implicated in carcinogenesis as well as maintenance of cancer stem cells. Somatic mutations in the components of Hh signaling (PTCH1 and SMO) have been shown to be a major cause of basal cell carcinoma, and dozens of Hh inhibitors have been developed. To date, two inhibitors (GDC-0449 and LDE225) have been approved by the U.S. Food and Drug Administration to treat basal cell carcinoma and medulloblastoma. Here, we review the role of the Hh signaling in the carcinogenesis and progression of gastric cancer and summarize recent findings on Hh inhibitors in gastric cancer. Hedgehog signaling is often aberrantly activated and plays an important role during inflammation and carcinogenesis of gastric epithelial cells. Further study of the precise mechanisms of Hh signaling in this disease is needed for the validation of therapeutic targets and evaluation of the clinical utility of Hh inhibitors for gastric cancer.

## Background

Gastric cancer (GC) is highly heterogeneous, largely because of the complex molecular mechanisms of its carcinogenesis (e.g., genetic alterations, epigenetic changes, infection, and interactions with the microenvironment) [1–3]. Consequently, existing targeted therapies, such as HER2 antagonist trastuzumab and VEGFR2 antagonist ramucirumab, are effective only in a small percentage of patients with GC. Therefore, more research is needed to develop effective personalized therapy for this disease [4]. In this context, the Hedgehog (Hh) signaling pathway is important to explore.

The Hh signaling pathway is crucial in embryonic development and tissue homeostasis [5–7]. Over the past decades, increasing evidence has also implicated aberrant Hh signaling in the initiation and progression of many cancers, including prostate [8], breast [9], pancreatic [10], and hepatocellular [11] cancers as well as GC [12]. In basal cell carcinoma (BCC) [13] and medulloblastoma [14] in particular, the role of Hh signaling has been well established. On the basis of these results, Hh signaling inhibitors have been developed, and two have been approved by the U.S. Food and Drug Administration (FDA) to treat BCC and medulloblastoma [15, 16]. However, the molecular mechanisms of the Hh signaling in other tumors appear far more complex than those in BCC. In addition to the canonical Hh signaling cascade, the crosstalk between components of Hh and other pathways contributes to carcinogenesis [17]. Moreover, Hh signaling is needed for the maintenance of cancer stem cells (CSCs) [18–20]. Thus, a deeper understanding of the role of Hh signaling in CSCs will provide a rationale for development of a druggable target to block metastasis and overcome chemotherapy resistance in GC.

In this review, we will focus on the role of the Hh signaling pathway in GC and CSCs and discuss the therapeutic potential of Hh inhibitors for GC.

### Hh signaling pathway overview

**Fig. 1:**
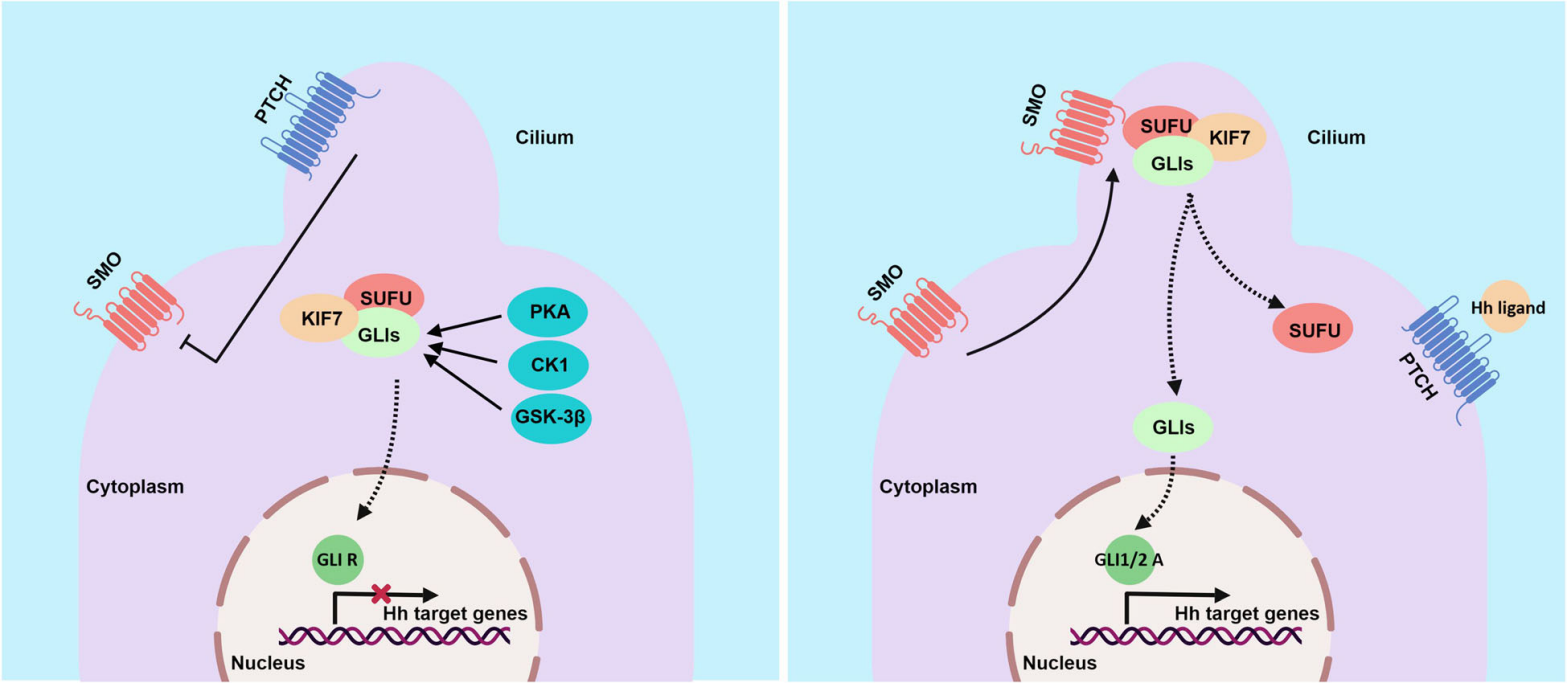
Hh signaling pathway transduction. **a** In the absence of Hh ligands, PTCH is located in the primary cilium and suppresses SMO activation by preventing SMO localization to and accumulation in the cilium. The GLI transcription factors are phosphorylated and processed into transcriptional repressors (GLI R) by several protein mediators (PKA, CK1, and GSK3β). GLI R translocates into the nucleus and inhibits transcription. **b** When an Hh ligand binds to PTCH, the inhibition of SMO is relieved, allowing dissociation of GLI transcription factors from KIF7 and SUFU. Transcriptional activators (GLI1/2 A) then enter the nucleus to induce expression of Hh target genes. Figure created with BioRender

The central components of the Hh signaling pathway are three Hh ligands (Sonic hedgehog [SHH], Indian hedgehog [IHH], and Desert hedgehog [DHH]), the transmembrane receptor Patched1 (PTCH1), the G-protein-coupled receptor Smoothened (SMO), the negative regulator Suppressor-of-fused (SUFU), and the transcription factors GLI1, GLI2, and GLI3 [7]. In general, Hh signaling is activated through the binding of the Hh ligand to PTCH1, which acts as a negative regulator of SMO [21]. The inhibition of SMO is then released, which activates a cascade that leads to the translocation of the downstream GLI transcription factors (GLIs) to the nucleus. Subsequently, the activated GLIs induce expression of various Hh target genes, such as BCL2, SNAI1, FOXM1, cMYC, and CCND1 [22]. In contrast, in the absence of Hh ligands, PTCH1 is located in the primary cilium and suppresses SMO activation by preventing its localization and accumulation in the cilium (Fig. 1).

GLIs play a central role in Hh signaling because abnormal activation of Hh signaling, whether due to mutations of pathway components or ligand-dependent or -independent mechanisms, triggers the downstream effector GLIs. Among the three GLIs, GLI1 acts exclusively as a transcription activator, while GLI2 and GLI3 act either as transcriptional activators or truncated transcriptional repressors [23]. Indeed, GLI1 was shown to activate the expression of genes involved in multiple cellular functions, including cell proliferation (cyclin D1/D2 and FOXM1) [24, 25], angiogenesis (VEGF family) [26], epithelial-mesenchymal transition (SNAI1) [27], and invasion (osteopontin and MMP) [28–30]. Furthermore, GLI1 exerts its function through crosstalk with several non-canonical Hh signaling, such as mTOR [31, 32], KRAS [33], TGFβ [34, 35], and WNT [36, 37]. Therefore, GLI1 is emerging as a promising target for blocking Hh signaling and treating cancer.

Broadly, the canonical mechanisms linking abnormal activation of the Hh signaling pathway to cancer can be ligand dependent or ligand independent. The best-known example of the ligand-independent mechanism is in BCC [13]. Somatic mutations in PTCH1 have been identified in more than 90% of sporadic BCC, and the dysfunctional PTCH1 leads to constitutively active SMO. While these tumors are insensitive to Hh ligands, small-molecule SMO inhibitors (e.g., cyclopamine) can effectively suppress these tumors’ growth. So far, BCC is the only tumor known to almost exclusively depend on mutation of Hh signaling pathway components. By contrast, mutations in PTCH1 and SMO are infrequent in other tumors and are rare in GC [38, 39].

**Fig. 2:**
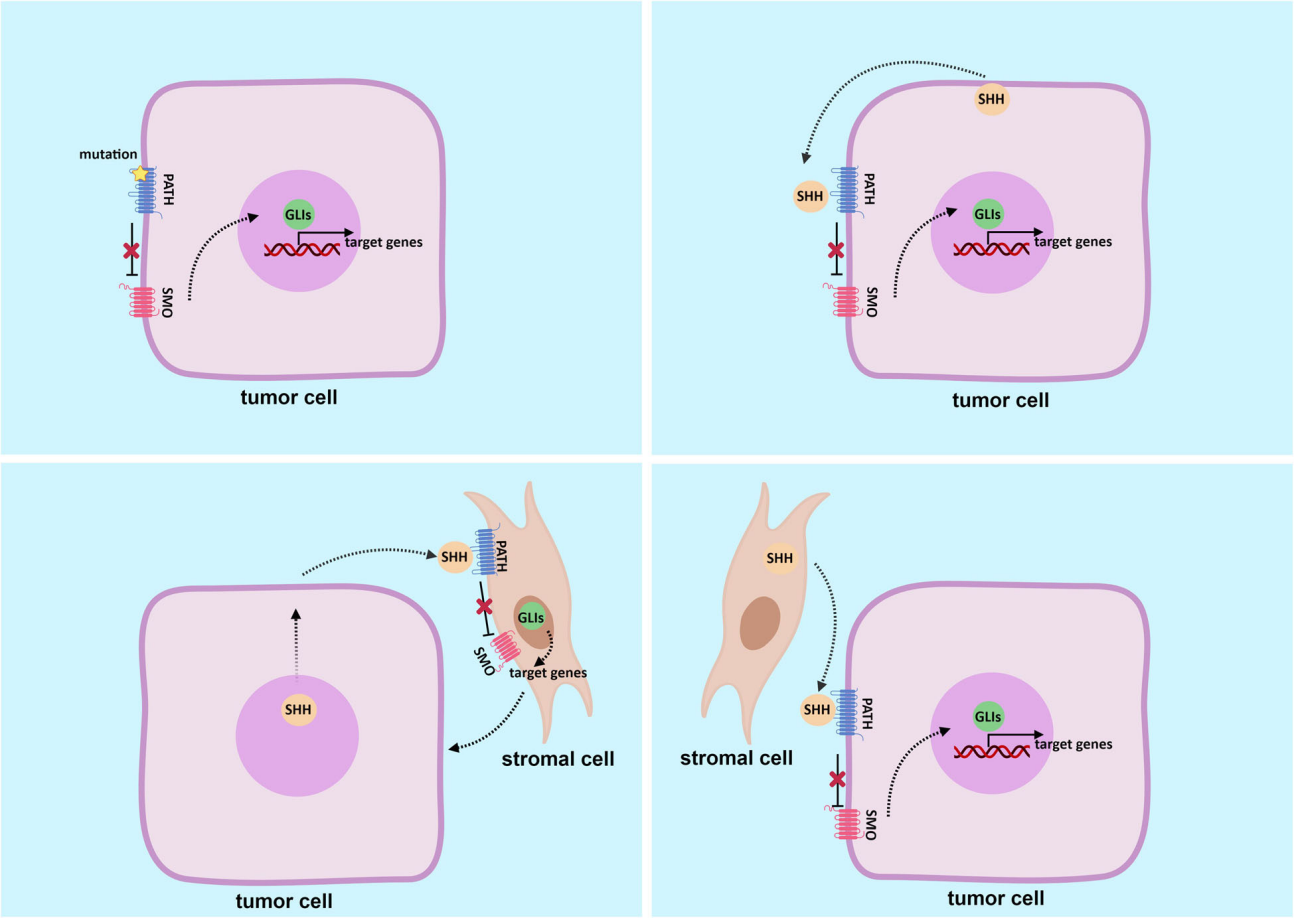
Mechanisms of Hh signaling pathway in cancer. **a** Ligand-independent mechanism: somatic mutations in PTCH1 or SMO lead to constitutive activation of Hh signaling. **b-d** Ligand-dependent mechanisms: autocrine regulation (**b**), paracrine regulation (**c**), and reverse paracrine regulation (**d**). Figure created with BioRender

On the other hand, the ligand-dependent mechanism has been observed in some gastrointestinal adenocarcinomas, e.g., pancreatic cancer and colon cancer [40]. This mechanism tends to implicate interaction between tumor cells and stromal cells in the tumor microenvironment. Thus, three modes of ligand-dependent regulation of Hh signaling have been proposed: autocrine regulation, in which a tumor-derived ligand activates Hh signaling in tumor cells; paracrine regulation, in which a tumor-derived ligand activates Hh signaling in stromal cells; and reverse paracrine regulation, in which a stromal cell–derived ligand activates Hh signaling in tumor cells [41] (Fig. 2). Finally, beyond these canonical mechanisms, the crosstalk between components of Hh signaling and other pathways contributes to carcinogenesis and progression in GC [42].

**Fig. 3:**
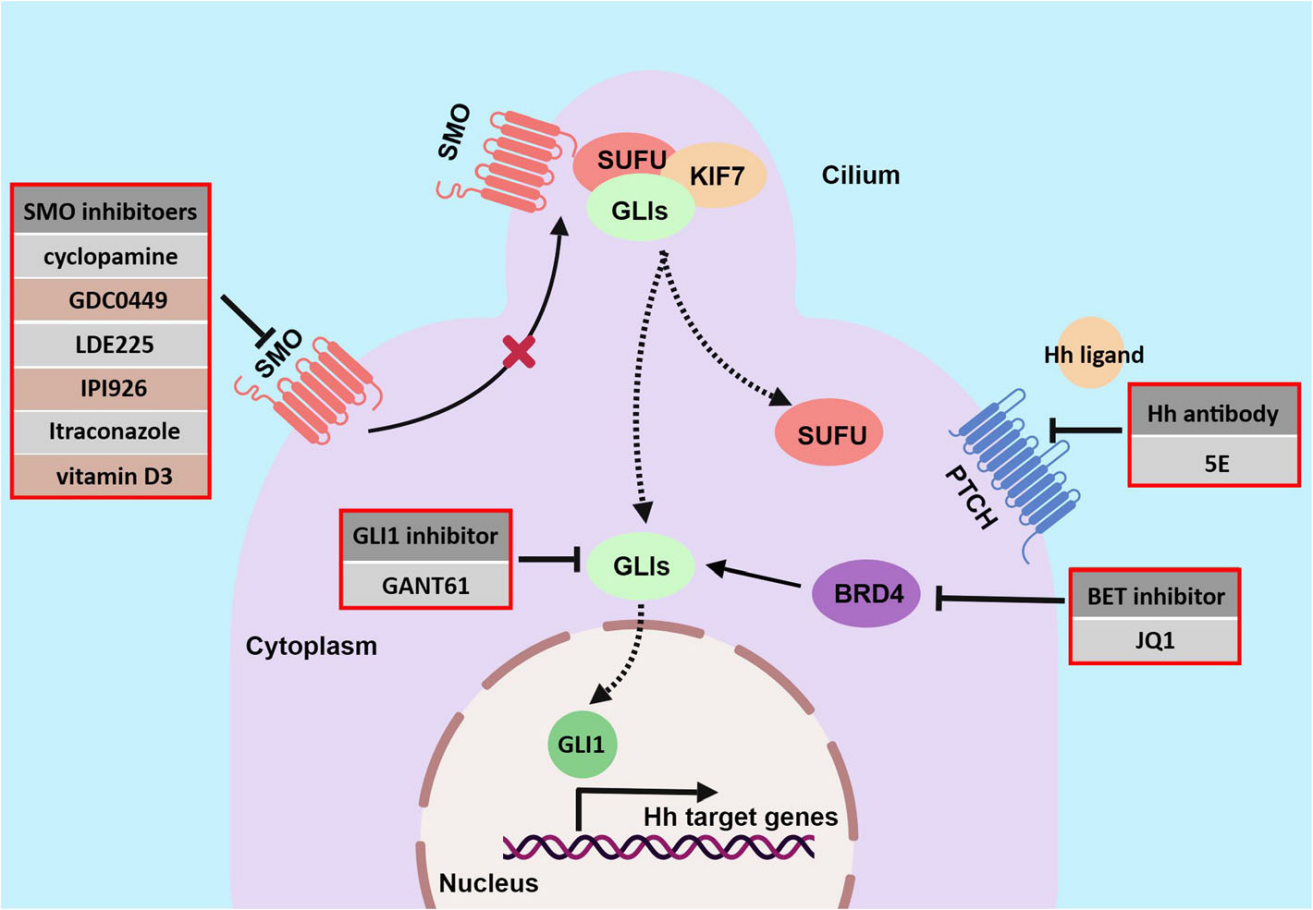
Targeting Hh signaling at different levels of the cascade. Hh ligand antibody, e.g., 5E1, blocks interaction of Hh ligand and receptor. SMO inhibitors, e.g., cyclopamine, directly bind to and inactivate SMO. GLI1 inhibitors, e.g., GANT61, interrupt transcriptional activity of GLI1 by directly binding to GLI1. BET inhibitor JQ1 represses Hh signaling by interfering with the regulation of BRD4 on GLI1/GLI2

### Hh signaling in GC

Hh signaling has been implicated as a critical factor in gastric gland organogenesis and differentiation during embryonic development. Although all three Hh ligands activate identical signaling cascades by binding to PTCH1, the distribution of these ligands exhibits tissue specificity. SHH and IHH are most highly expressed in the gastrointestinal tract [43], while DHH expression is restricted to testes and the nervous system [44]. In the adult stomach, the Hh pathway not only regulates gastric epithelial cell differentiation and maturation [45, 46] but also is essential to the physiology of the stomach [47, 48]. Hh ligands (typically SHH in the stomach) secreted by the epithelial cells are recognized by receptors on stromal cells, which initiates the Hh signaling cascade in stromal cells and increases transcription of downstream target genes. In turn, these genes’ products are involved in maintaining the microenvironment of the epithelium. It is so-called “paracrine” regulation [49].

In precancerous lesions in the stomach, the balance of this “paracrine” regulation is broken, which results in metaplastic transformation and growth of the fibrous tissue, proceeding to carcinogenesis. Interestingly, in the chronic inflammatory setting, e.g., *Helicobacter pylori* infection, expression of SHH is downregulated in inflamed tissues [50, 51], mainly because of the loss of parietal cells and epithelium atrophy [52]. However, with gastric lesion progression, increasing expression of SHH is accompanied by epithelial regeneration and proliferation [53]. These observations underline the important role of SHH and Hh signaling in gastric epithelial repair and regeneration [54]. Furthermore, GC cells show not only elevated SHH expression but also increased PTCH1 receptor expression [55]. Thus, excess SHH stimulates Hh signaling and promotes GC cell proliferation and progression. In the latter case, besides “paracrine” regulation, “autocrine” regulation also contributes to the progression of cancer.

Previous studies demonstrated that the overactivity of Hh signaling is a common molecular event in GC and that this abnormal activity is blocked by Hh inhibitors (e.g., cyclopamine) and Hh antibodies [12, 56]. In addition, a number of studies showed that overexpression of SHH is associated with unfavorable clinical outcomes (e.g., advanced clinical stage, lymph node metastasis, and poor prognosis) in patients with GC [57]. Altogether, these results suggest that the Hh signaling pathway participates in cell migration and metastasis. Furthermore, the insulin-like growth factor/phosphoinositide 3-kinase (PI3K)/Akt pathway shows a reciprocal relationship with Hh-dependent tumor formation during GC cell migration. Yoo et al. reported that the Hh pathway promotes GC progression and metastases through activation of the PI3K/Akt pathway [58]. Akt, in turn, stabilizes full-length GLI2 through phosphorylation of S230, thereby amplifying the transcriptional output of Hh signaling [59]. This evidence not only confirms the role of Hh signaling in gastric carcinogenesis and progression but also raises the possibility of inhibition of Hh signaling for treatment of GC.

### Hh signaling, CSCs, and drug resistance

Abundant evidence indicates that Hh signaling is involved in the maintenance of CSCs in many cancers [18–20]. Components of Hh signaling have been found to be specifically overexpressed in subpopulations of cancer cells with CSC properties. Moreover, these putative CSCs, such as those in pancreatic cancer (ALDH+ cells), colon cancer (CD133+ cells), breast cancer (CD44 + CD24− cells), and GC (CD44+ cells) are sensitive to Hh inhibitors [60–63]. For example, Yoon and colleagues found enrichment of CD44 along with increased levels of Hh pathway components and certain self-renewal marker proteins (SOX2, OCT4, and NANOG) in three GC cell lines [64]. In these GC lines, Hh inhibition with SMO shRNA or small-molecule inhibitors significantly suppressed spheroid formation and tumor growth. Furthermore, while CD44+ spheroid cells were highly resistant to chemotherapy (5-fluorouracil and cisplatin), this chemoresistance was reversed with Hh inhibition.

To date, the molecular characterization and functional relevance of CSCs in solid tumors are not well understood. Nevertheless, the close relationship between Hh signaling and CSCs raises the possibility of the combination of an Hh inhibitor and standard chemotherapy to improve antitumor efficacy. To achieve the goal, the precise molecular mechanisms of CSCs with Hh signaling need to be further investigated. Meanwhile, from a technical viewpoint, at least two challenges need to be resolved. First is the identification of reliable biomarkers to distinguish CSCs and to predict benefit from therapy. The baseline expression of Hh ligands in tumor tissue appears not to provide a positive association between clinical benefit and high activation of Hh signaling from treatment with Hh inhibition [65]. Instead, CSC-related biomarkers should be considered potential candidates for patient selection. Second is identifying which component of Hh signaling would be an ideal target. To date, different types of inhibitors have been developed that target multiple signal transduction elements of the Hh signaling cascade. However, most of these inhibitors have failed to show the desired results in clinical trials for GC.

### Hh inhibitors and clinical potential in GC

Considering the critical role of the Hh pathway in carcinogenesis, targeting of Hh signaling has attracted substantial interest. So far, dozens of small-molecule inhibitors of Hh have been developed, and two (GDC-0449 and LDE225) have been approved by the FDA for treating BCC and medulloblastoma [15, 16], but none have been approved for GC. Here, we focus on the inhibitors that have been investigated in GC (Table 1, Fig. 3).

**Table 1:** Summary of Hh signaling antagonists studied in gastric cancer

Target	Compound	Type	Mechanism of action	Study status	Clinical trial	References
Hh ligand	5E1	monoclonal antibody	Block SHH protein activity	In vivo	N/A	[66–68]
SMO	cyclopamine	Small molecule inhibitor	Bind to SMO protein heptahelical bundle and inhibit SMO activity	In vivo	N/A	[10, 55, 69–71]
	GDC-0449 (Vismodegib, Erivedge)	Small molecule inhibitor	Bind to the extracelluar domain of SMO	In vivo	NCT00982592	[64, 72, 73]
	IPI-926 (Saridegib)	Small molecule inhibitor	derivative of the cyclopamine, antagonist of SMO	In vivo	N/A	[74]
	Itraconazole	Small molecule inhibitor	Bind to SMO protein	In vivo	N/A	[75]
	Vitamin D_3_	Small molecule inhibitor	Bind to SMO protein	in vitro	N/A	[76]
GLI1	GANT61	Small molecule inhibitor	bind GLI1 protein between zinc finger 2 and 3	In vivo	N/A	[63, 77]
BRD4	JQ1	Small molecule inhibitor	Inhibit BRD4 binding to GLI	N/A	N/A	[73]

#### Hh ligand antibody

The anti-SHH monoclonal antibody 5E1 blocks Hh signaling by binding at the SHH pseudo-active site groove [78]. 5E1 has been used as a research tool to study Hh signaling under physiologic or pathologic circumstances. A previous study showed that mesenchymal stem cells are recruited from bone marrow and contribute to a tumor niche that promotes and sustains GC progression [79]. Blocking Hh signaling using 5E1 significantly inhibited the proliferative response of mesenchymal stem cells to the cytokine interferon-gamma [66]. These data support a synergic interaction of Hh signaling and cytokines during precancerous lesions.

#### SMO inhibitors

Cyclopamine is the first small-molecule inhibitor of Hh signaling, extracted from corn lilies, and it inactivates SMO by directly binding to its heptahelical bundle [80, 81]. Cyclopamine has been found to effectively inhibit Hh and tumor growth in vivo and in vitro [55, 69–71]. However, the off-target effects that accompany a high concentration of cyclopamine require careful evaluation to avoid false-positive data. Moreover, owing to its severe toxicity and low oral bioavailability, cyclopamine is not a suitable drug [82]. A recently developed derivative of cyclopamine, IPI-926 (also called saridegib), showed improved properties compared with cyclopamine [83]. In preclinical studies, IPI-926 was a reliable inhibitor of tumor growth and overcame chemoresistance in a number of cancers [74, 84, 85]. Based on these results, several phase I and phase II clinical trials in pancreatic cancer, head and neck cancer, and other cancers have been conducted. However, to date, detailed results remain to be disclosed.

GDC-0449 (vismodegib), which acts by binding to the extracellular domain of SMO and antagonizing Hh signaling, has shown promising anti-tumor activity in advanced BCC and became the first Hh signaling inhibitor approved by the FDA in 2012 for the treatment of metastases and locally advanced BCC [15, 16]. Although GDC-0449 significantly suppressed tumor proliferation and invasion in vivo and in vitro [72, 86, 87], no satisfactory results have been obtained from clinical trials in GC so far [65]. In a randomized phase II study (NCT00982592), GDC-0449 in combination with chemotherapy was investigated in advanced GC. There was no difference in response rate or survival time with the addition of GDC-0449 to chemotherapy. However, in the patients given chemotherapy combined with GDC-0449, those with higher CD44 expression tended to fare better [64]. In contrast, in the chemotherapy-only group, patient survival was not stratified by CD44. As mentioned above, an accurate biomarker is necessary to identify a subpopulation that could benefit from Hh inhibition.

Other SMO inhibitors, including LDE225 (sonidegib), PF-04449913 (glasdegib), itraconazole, LEQ506, BMS-833923, LY2940680, and vitamin D3, have been effective against a variety of human cancers [88–90], and some have reached clinical trials. These agents are being investigated as potential treatments for hematologic malignancies, medulloblastoma, and other solid tumors, but not for GC [91, 92].

#### GLI1 inhibitors

Since the GLI1 transcription factor is the final effector of the Hh signaling cascade, targeting GLI1 may yield more efficient antitumor activity than other targets have thus far. In 2007, two small-molecule GLI1 inhibitors (GANT58 and GANT61) were identified through cell-based screening [93]. GANT61 directly binds GLI1 protein between zinc fingers 2 and 3 and interrupts GLI1 binding to target DNA, thereby inhibiting transcription [94].

Yan and colleagues treated GC cell lines with cyclopamine and GANT61 and found that both inhibitors repressed cell growth [77]. Another study, by Xu et al., showed that GANT61 increased apoptosis of CD44+/Musashi-1+ GC cells [63]. The study also demonstrated a synergistic effect between the Hh signaling inhibitor and the chemotherapy drug doxorubicin. Thus, GANT61 shows therapeutic potential, and further in vivo study and clinical trials are required to investigate the antitumor activity of GANT61 in GC.

Besides direct GLI1 inhibitors, indirect GLI1 inhibitors have also recently emerged as candidates for suppressing Hh-dependent tumors. JQ1, the bromodomain and extra-terminal domain (BET) protein inhibitor, specifically impacts GLI1 transcription [73]. BET proteins act as a transcriptional regulator by binding to chromatin during mitosis, promoting cell cycle progression. BET proteins also form multiprotein complexes with the positive transcription elongation factor b, activate RNA polymerase II, and enhance gene expression [95]. Recently, Tang and colleagues demonstrated that BRD4, a member of the BET family, is the critical regulator of GLI1/GLI2 by occupying their promoters and modulating transcription [73]. Furthermore, JQ1 effectively suppresses Hh-dependent tumor growth by preventing BRD4 from binding to GLI1/GLI2. Even more encouragingly, JQ1 impaired GLI signaling in the setting of acquired resistance to SMO inhibitors. These results suggests a novel strategy for the treatment of Hh-dependent tumors using BET inhibition. Previous studies demonstrated that JQ1 efficiently suppressed proliferation and induced apoptosis of GC cells through BRD4 and downstream genes [96, 97]. However, no study on the effect of JQ1 on Hh/GLI1 signaling in GC has been reported. Interestingly, in certain GC cell lines with low expression of BRD4 or c-MYC (a BRD4 target protein), the antitumor activity of JQ1 was still remarkable [98]. This finding suggests that other molecular mechanisms are involved in this process.

### Perspective

The Hh signaling pathway has long been thought to play a crucial role in embryonic development, tissue homeostasis, carcinogenesis, and maintenance of CSCs. Since the predominant role of Hh signaling in BCC has been verified, leading to the development and marketing of vismodegib, efforts are underway to exploit this pathway to treat other cancers. However, so far, the results from clinical trials targeting Hh signaling in a number of cancers, such as GC, colorectal cancer, ovarian cancer, and pancreatic cancer, have been inconsistent with expectations [64, 65, 99, 100]. The explanation for these results is likely that other genes or signaling pathways are also involved in carcinogenesis and progression.

The reasonable way to overcome this problem is the identification of precise predictive biomarkers and optimized combination strategies, such as an Hh signaling inhibitor combined with standard chemotherapy or other targeted therapy. In one study of Hh signaling in medulloblastoma, Shou and colleagues validated a five-gene signature that identifies Hh pathway activation and patients most likely to respond to therapy targeting the Hh signaling pathway [101]. Although the panel is not yet mature and needs to be improved [102], it still broadens our research strategy for identifying better predictive and prognostic markers. Furthermore, given the crucial role of Hh signaling in the maintenance of CSCs, CSC-related proteins could be used to select patients who are likely to benefit from an Hh inhibitor. Indeed, several proteins or panels have been proposed as CSC biomarkers in GC [103–111], such as ALDH1, CD24/CD44, CD54/CD44, EPCAM/CD44, LGR5, CD90, and CD133. Thoroughly understanding the mechanisms of CSCs and Hh signaling will provide rationales for more precise approach.

## Conclusions

Hh signaling is often aberrantly activated and plays an important role in the inflammation and carcinogenesis of gastric epithelial cells. However, the clinical utility of Hh inhibitors for GC should be further evaluated through more well-designed clinical trials. In addition, given the complexity of Hh signaling and the heterogeneity of GC, the precise mechanisms of Hh signaling need to be studied further for the validation of therapeutic targets and ideal biomarkers.

## Data Availability

Not applicable.

## Abbreviations

BCC: basal cell carcinoma
BET: bromodomain and extra-terminal domain
CSC: cancer stem cell
DHH: Desert hedgehog
FDA: U.S. Food and Drug Administration
GC: gastric cancer
GLI: glioma-associated oncogene
Hh: Hedgehog
IHH: Indian hedgehog
PTCH1: Patched1
SHH: Sonic hedgehog
SMO: Smoothened
